# Perfluorinated Carbon Chain Length Drives Uptake of Diverse Per- and Polyfluoroalkyl Substances in Field-Deployed Passive Samplers

**DOI:** 10.1021/acsestwater.4c01164

**Published:** 2025-03-07

**Authors:** Matthew Dunn, Jitka Becanova, Simon Vojta, Heidi Pickard, Rainer Lohmann

**Affiliations:** † Graduate School of Oceanography, 4260 University of Rhode Island, Kingston, Rhode Island 02882, United States; ‡ Harvard John A. Paulson School of Engineering and Applied Sciences, 1812 Harvard University, Cambridge, Massachusetts 02138, United States

**Keywords:** per- and polyfluorinated alkyl substances, passive sampler, aquatic contamination

## Abstract

Per- and polyfluoroalkyl substances (PFAS) are a group of compounds of high concern due to their ubiquity, persistence, and adverse health impacts. With a diversity of chemical structures and properties, detection tools are needed to capture as many PFAS as possible. In this study, a microporous polyethylene tube (MPT) passive sampler was calibrated for 25 target compounds, 8 suspect PFAS, and extractable organofluorine (EOF) during 1–2 week deployments in groundwater, freshwater river, and estuary contaminated by aqueous film-forming foam (AFFF). Targeted analysis, suspect screening, and EOF were performed on passive and grab samples to derive sampling rates, *R*_s_. Median measured and fluorine-normalized estimated EOF *R*_s_ in groundwater (7.1 vs 8.4 mL day^–1^ respectively) and river water (55 vs 66 mL day^–1^ respectively) were within 20% of each other. For suspect PFAS, *R*_s_ were similar to targeted PFAS of alike functional group chemistry and perfluorinated carbon chain length. For example, for 6:2 and 8:2 FTSAS-sulfoxide, estimated *R*_s_ values were 1.8 and 6.0 mL day^–1^, respectively, similar to *R*_s_ measured for 6:2 and 8:2 FTS of 2.2 and 6.3 mL day^–1^. These results suggest that targeted and suspect PFAS and EOF are predictably taken up by MPT samplers, expanding passive sampling capabilities.

## Graphical abstract

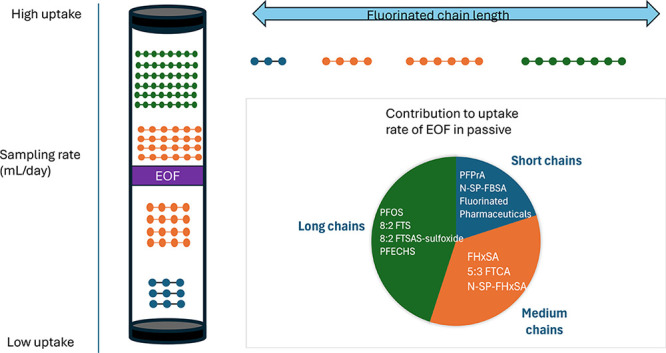

## Introduction

Per- and polyfluoroalkyl substances (PFAS) are a family of contaminants of great concern due to their ubiquity, persistence, and adverse human health impacts.^1,2^ PFAS can be difficult to measure in environmental matrices, given their diversity in chemical structures, physicochemical properties, range in environmental concentrations, and expected natural fluctuations in the environment. Thus, passive sampling has emerged as a preferable approach to monitor PFAS in surface and groundwaters across temporal, spatial, and climatic conditions.^3–7^ In recent years, progress has been made in designing, calibrating, and applying passive samplers for PFAS detection across both equilibrium and integrative regimes.^6,8–13^ This work has mostly focused on a limited number of PFAS amenable to detection through targeted analysis, such as legacy PFAS groups comprising perfluoroalkyl carboxylates (PFCA) and perfluoroalkyl sulfonates (PFSA), as well as precursor groups to PFSAs and PFCAs, such as perfluoroalkyl sulfonamides (FASA) and fluorotelomer sulfonates (FTS).^8–11,13^

However, validating the performance of integrative passive samplers for a wider range of diverse PFAS that lack analytical standards has yet to be achieved. The aim of this work is to close this gap by focusing on PFAS present in the environment due to the use of aqueous film-forming foams (AFFF).^14–18^ AFFF were widely applied at airports and military bases for firefighting and fire training exercises and represent a significant ongoing source of PFAS to aquatic ecosystems and drinking water supplies.^18–23^

Significant effort has been put into modeling integrative passive sampling mass transport for PFAS and other polar organic compounds of concern, specifically for the microporous polyethylene tube (MPT) passive sampler.^5,8,24–27^ The MPT design features a durable, thick membrane that allows for passive sampling in harsh environmental conditions, while offering flow-resistant uptake.^8,10,26^ These models aim to predict the sampling rate, *R*_s_, which is needed to convert passive sampling-accumulated amounts (of PFAS) to their time-weighted average concentrations.^8,24^ These efforts require an understanding of individual PFAS diffusivity and sorption kinetics, currently limited to well-studied compounds.^8,13,28^ To better assess PFAS concentrations beyond the limited suite of targeted compounds with available analytical standards, techniques such as suspect screening and extractable organofluorine analysis (EOF) are increasingly used.^29–36^ This is especially true for environmental waters impacted by AFFF.^14–17,19–23,37^ EOF analysis provides a proxy for total PFAS by capturing the amount of extractable organofluorine present in a system, which may include fluorinated pharmaceuticals and pesticides, in addition to targeted PFAS.^38,39^ Suspect screening of nontargeted PFAS has been used to expand the list of PFAS analytes that can be identified without the use of registered analytical standards.^30,31,40–42^

Major progress has been made in conveying confidence in the identification of suspect compounds using high-resolution mass spectrometry.^29,30^ The data produced by suspect screening methods are commonly presented qualitatively, limiting their utility in risk-based assessments.^40^ Some studies have adopted a calibration curve using commercially available standards with comparable molecular structures, functional groups, and ionization behaviors to semiquantify suspect compounds.^43–45^ More recently, a global correction or global calibration curve has been proposed, employing an average calibration curve for all targeted compounds.^41,46^ This method is particularly advantageous when dealing with a large number of structurally diverse compounds, as it streamlines data processing while still providing reasonably accurate estimates of concentrations. Each of these approaches has its limitations, and it is difficult to assess the uncertainty associated with semiquantifying PFAS using these approaches. The influence of the matrix on diverse compounds during sample preparation and ionization efficiencies and detector responses across various chemistries provide an inherent bias that cannot be easily quantified within these calculations.^42^ We chose to compare these different approaches for estimating concentrations to assess the variability in deriving *R*_s_ for suspect PFAS without available analytical standards.

In summary, the objectives of this study were to (i) compare the uptake rates of an MPT passive sampler in different aquatic environments, (ii) derive sampling rates for EOF and AFFF-derived PFAS identified via suspect screening, (iii) assess whether sampling rates for suspect compounds are best approximated from molecular weight, fluorinated chain length, or functional group, and (iv) compare different approaches for deriving sampling rate from suspect screening results.

## Methods

### Chemicals and Reagents

Liquid chromatography-grade methanol and water were purchased from Fisher Scientific (New Hampshire, USA) along with ammonium hydroxide, ammonium acetate, ACS-grade ethanol, and ACS-grade methanol. Analytical standards were used, including native compound standards from the Wellington PFAC-30PAR mix plus an additional four analytical compounds (Table S1). Mass labeled surrogate solutions were derived from Wellington Laboratories’ MPFAC-24ES, and an additional three mass labeled compounds were purchased from Wellington Laboratories (Canada) (Table S1).

### Passive Sampler Assembly and Extraction

Passive samplers were assembled as described previously using 7 cm long porous high density polyethylene membranes (HDPE) and approximately 600 mg of hydrophilic lipophilic balance (HLB) sorbent.^8,10^ Passive samplers were extracted for target and suspect PFAS using sequential LC-grade methanol solid–liquid extractions spiked with mass-labeled surrogates for quantification and recovery assessment, based on previously published methods.^8,10^ For EOF extraction, slight modifications were made in which mass labeled surrogates were not added prior to extraction, and passive samplers underwent a 24-h rinse with 9 mL of 0.01% ammonium hydroxide in water (volume/volume) prior to the methanol extractions, with no addition of ENVI-carb cleanup.^21^

### Field Deployments of Passive Samplers

Passive sampler replicates were deployed for 1–2 weeks at 3 sites in Cape Cod, Massachusetts, during the summer of 2022. The short-term deployments were based on prior results that showed most PFAS exiting the linear uptake by MPT samplers after approximately 2 weeks.^8^ Weekly deployments were chosen for the groundwater well at a previously studied fire training area at the Joint Base Cape Cod (Site 1) due to high concentrations of PFAS that may saturate the MPT sampler during longer sampling intervals. These deployments were conducted from July 20th to August 10th at the previously characterized site.^17,18^ 2-week deployments were conducted from July 6th to August 18th, 2022 at previously studied downstream sites in the freshwater Quashnet River (site 2) and at the entrance of the Quashnet River into Waquoit Bay’s tidal estuary (site 3).^21^ At each site, passive samplers were deployed for targeted/suspect screening analysis with separate replicates for EOF analysis. Recovered passive samplers were frozen at −4 °C prior to extraction and analysis. Additional details on site collection are provided in Table S2.

Water grab samples (500 mL) were collected at site 1 (*n* = 2) and from sites 2 and 3 (*n* = 4 each) using solvent-rinsed high density polyethylene bottles. Grab samples were collected at the deployment and collection of each set of passive samplers at sites 1–2 for both targeted and EOF analyses and were stored frozen at −4 °C prior to extraction and analysis. Field blanks for both grab samples (*n* = 2) and passive samplers (*n* = 3) were taken at all three sites.

### Grab Sample Extraction

Samples of 50–500 mL were preconcentrated using a solid phase extraction method described in previous studies.^8,10,11,47^ Site 1’s samples were extracted as 50 mL subsamples due to high levels of PFAS. For most samples from sites 2–3, the 500 mL grab sample was split into two 250 mL halves, with one portion being filtered through a glass fiber filter (Whatman GF/C 47 mm diameter) and the other remaining unfiltered. For additional details on the filtration approach, see the Supporting Information, and matrix spike results are given in Table S3.

### Targeted and Suspect Screening Analysis

The instrumental analysis was performed using an SCIEX Exion LC AC UHPLC system coupled to a SCIEX X500R quadrupole time-of-flight tandem mass spectrometer (QTOF MSMS). For the quantification of the target PFAS and fluorinated pharmaceutical analytes, an HRMS/MS (MRM HR) method was used. The negative ESI with the following parameters was used: curtain gas at 30 psi, ion source gas 1 at 40 psi, ion source gas 2 at 60 psi, and temperature 450 °C (see Table S1 for compound-dependent parameters). The MS data for the suspect screening analysis were collected using Sequential Window Acquisition of All Theoretical Mass Spectra (SWATH) mode with the ion source operated under the same conditions. The targeted analysis and suspect screening analysis resulted in two distinct data sets used in this study. For additional details, see the Supporting Information.

### Extractable Organofluorine Analysis

Samples were measured for EOF at Harvard University following established methods using a combustion ion chromatograph (CIC) with a combustion unit from Analytik Jena (Jena, Germany) and a 920 Absorber Module and 930 Compact IC Flex ion chromatograph from Metrohm (Herisau, Switzerland).^39^ Sample extracts (100 μL) were injected into the combustion unit at 1050 °C, and the anions were separated with an ion exchange column (Metrosep A Supp 5-150/4) operated at 30 °C, with sodium carbonate–bicarbonate buffer as the eluent and isocratic elution. The fluorine concentration was measured via ion conductivity.

### Quality Assurance and Quality Control

For the targeted analysis data, method detection limits (MDLs) were calculated from laboratory and field blanks collected for both passive sampler and solid phase extraction procedures. The median and three times standard deviation of the blank concentrations were summed to determine MDLs (see Table S4 for passive and grab samplers MDLs). Method recovery was evaluated using mass labeled surrogate standards (Table S5). Acceptable recoveries of mass labeled standards ranged from 40 to 140%; passive sampler and solid phase extraction results were not reported for any compounds or samples with recoveries outside of this range.

The identification of PFAS using the suspect screening approach was performed using the SCIEX OS software and was based on precursor mass, isotope pattern, retention time, exact mass accuracy (<5 ppm), and MS/MS fragmentation matching (SCIEX Fluorochemical HR-MS/MS Spectral Library 2.0 Activ). All the identified compounds were assigned the confidence level of identification 2a (MS/MS spectral library match), following the conventions from Schymanski et al. and Charbonnet et al.^29,30^

For EOF analysis of passive samplers and grab samples, method validation was performed, as detailed in the Supporting Information. EOF concentrations above the instrumental background were corrected based on extraction blanks. MDLs were calculated based on average concentrations in extraction blanks plus three times the standard deviation adjusted by each sample’s dilution factor. Results of reproducibility, organofluorine recovery, and inorganic fluorine removal are detailed in the Supporting Information.

### Determination of Sampling Rates

Sampling rates were derived for each individual compound/EOF using eq 1.

$$R_{s}=\frac{m_{\mathrm{ps}}}{C_{w}\;\times t}$$ 1

where *R*_s_ is the sampling rate (mL day^–1^), *m*_ps_ is the mass (ng) of each targeted compound, suspect compound, or EOF detected in a sampler, *C*_w_ is the average measured water concentration (ng mL^–1^) from grab samples taken at deployment and collection of the passive sampler, and *t* is the field-deployment time (days).

For additional sampling rate details and notes on approaches, see the Supporting Information.

### Estimation of Sampling Rates for PFAS Without Analytical Standards

The sampling rate, or *R*_s_, for suspect compounds was determined using eq 1, while the analyte mass was replaced with the peak area for a subset of 8 samples that were run for suspect screening. These samples included one passive sampler for each site and both filtered and unfiltered water grabs from each site, when applicable. Sampling rates were calculated in this manner for both suspect and target compounds to allow for a comparison. Peak areas with no correction (*R*_s,area_), peak areas divided by a corresponding surrogate peak area (*R*_s,surrogate_), and peak areas divided by the average surrogate peak area (*R*_s,global_) were all used to calculate sampling rates for method comparison.

The use of an uncorrected area, while normally avoided, was included to perform a comparison to sampling rates for EOF data. This is done to limit bias from the use of mass labeled surrogate standards, which are not used in quantitative EOF analysis, thus following the practices outlined for comparing targeted data to EOF from literature.^39^ The *R*_*s*_ calculated from the peak area (*R*_s,area_) for each target and suspect compound was then divided by the number of fluorine atoms in each compound as a method of normalizing, henceforth referred to as *R*_s,Fnorm_ (eq 2). In eq 2, an example of this calculation is provided using two short chain PFAS as examples: perfluorobutanoic acid (PFBA) and perfluoropentanoic acid (PFPeA). The sampling rate of PFBA is then divided by the number of fluorine atoms in PFBA (C_4_HF_7_O_2_). This calculation was done for the one passive sampler per site that was also analyzed using suspect screening so that suspect compounds could be included in the estimation. Thus, all estimated *R*_s,fnorm_ values are based on *n* = 1.

$$R_{s,\mathrm{Fnorm}}=\Sigma \left(\frac{R_{s,\mathrm{PFBA}}}{7},\frac{R_{s,\mathrm{PFPeA}}}{9},\cdot \cdot \cdot .\right)$$ 2

The sum of these normalized sampling rates (*R*_s,Fnorm_) was then compared to mean EOF sampling rates. A complete summary of all sampling rate calculations and labels can be found in Table 1.

**Table 1:** Overview and Explanation of Sampling Rate Terms Discussed in This Study

sampling rate label	description	calculation
*R*_s,area_	using uncorrected peak areas for comparison to EOF data	eq 1
*R*_s,surrogate_	using peak areas corrected for internal surrogate of similar chain length	eq 1
*R*_s,quant_	using measured, quantified concentrations	eq 1
*R*_s,EOF_	using measured EOF concentrations	eq 1
*R*_s,Fnorm_	using *R*_s,area_ normalized for fluorine content	eq 2

### Uncertainty Calculation for Resulting Sampling Rates

To attempt to estimate uncertainty associated with these resulting sampling rates, the percent difference between each novel sampling rate approach and the quantitative target analysis-derived sampling rate (*R*_s,quant_) were evaluated. The sampling rates calculated from raw peak abundance (*R*_s,area_), from surrogate correction using a similar target compound (*R*_s,surrogate_), and using a global correction approach (*R*_s,global_) were all calculated for targeted PFAS analytes such as perfluorooctanoic acid (PFOA) or perfluorooctane sulfonic acid (PFOS). Thus, the bias in the sampling rate calculation for each method can be preliminarily estimated by comparing it to the quantified sampling rate of a target compound.

This calculation of uncertainty was applied to compare the impact of using filtered versus unfiltered water as well as to compare uncertainty between representative PFAS groups including PFCA, PFSA, FTS, and FASA. We assigned the mean uncertainty of the PFAS class most like the suspect compound being evaluated, such as the sampling rate of N-sulfopropylperfluorohexanesulfonamide (N-SP-FHxSA), which is assigned the mean uncertainty of all FASA compounds sampling rates (see Table S6 for details).

## Results

### Grab Water Samples

Thirty compounds were detected at least once in grab samples via targeted analysis, with a large range in concentrations for individual compounds (0.02–4400 ng L^–1^) across the three different sites (Table S7). This was expected based on the highly contaminated groundwater at site 1, a former fire training area, compared to the diluted downstream surface waters of the river and estuary. Several PFCAs were detected in every grab sample analyzed, ranging from 2.3 to 835 ng L^–1^ per compound. Additionally, many PFSAs were also ubiquitously detected, at 0.94–4400 ng L^–1^. Concentrations of all compounds were 2 orders of magnitude higher in the groundwater than in the surface waters of the river and estuary. For example, perfluorohexane sulfonate (PFHxS) and perfluorooctane sulfonate (PFOS) ranged from 280 to 4400 ng L^–1^ in groundwater compared to 2.6–44 ng L^–1^ in the river and estuary. No fluorinated pharmaceuticals were detected in the grab water samples based on targeted analysis (Table S1).

On average, the PFAS concentration in unfiltered water was 31% higher than the filtered water concentrations at site 2 (river) and 2.2% lower at site 3 (estuary). This discrepancy may be related to differences in total suspended sediment concentrations between sites (which were not determined at the time). These average values are biased high by the discrepancy between FASA concentrations in filtered water versus unfiltered water. In the river, there was a 200% difference in FASA concentrations when the water was unfiltered and a 38% difference in FASA concentrations when the water was unfiltered in the estuary. Concentrations of FASA, specifically FHxSA, were lower in the estuary than in the river (3 vs 24 ng L^–1^). This suggests FASA may be sensitive to filtration due to partitioning to particles, and this should be considered when estimating sampling rates for passive samplers that only collect the dissolved (not particle-bound) phase of PFAS. For reference, PFCA and PFSA showed negligible difference between filtered and unfiltered water at both river and estuary sites (0.55–6.0% difference). Thus, for calculating targeted sampling rates from quantified data, all water samples were grouped regardless of filtering status except for the FASA, which used filtered water concentrations.

EOF results for grab samples displayed a trend similar to that of targeted PFAS for groundwater and river samples, ranging from 189 to 276 in the river and from 5670 to 8830 ng F L^–1^ in the groundwater (Table S8). Groundwater at site 1 near the fire training area was elevated with an average EOF concentration of 7200 ± 2200 ng F L^–1^ (Table S8). The identification of suspect PFAS (Table S9) followed these site trends with a greater number of suspect compounds with greater peak area abundance identified at site 1 (8 compounds) compared to sites 2 and 3 (5 compounds, which were also detected at site 1). Sites 2–3 were generally similar in suspect compound presence, but unfiltered and filtered suspect abundances differed slightly (Table S10). For additional discussion of filtered versus unfiltered water results, see Supporting Information.

### Passive Sampler Results

32 targeted PFAS were detected in passive samplers at least once. Amounts ranged from 0.01 to 130 ng sampler^–1^ across the three sites, with the lowest mass uptake in the estuary and the highest in the groundwater (Table S11). Site 1’s groundwater deployments displayed significantly higher masses of PFAS in the samplers, despite a shorter deployment length (7 days) compared to sites 2–3 (14 days). PFAS commonly associated with AFFF plumes, including PFHxS, PFOS, FHxSA, FOSA, and 8:2 FTS, were orders of magnitude higher in site 1 passive samplers relative to passive samplers deployed at the river and estuary sites.^20^ While grab water concentrations were similar between sites 2 and 3, passive sampler results showed an average of 4 times higher mass of PFAS in the river passive samplers than the estuary passive samplers, for the same 14-day deployments (Table S11). Several compounds were detected in every passive sampler analyzed, including PFPeA, PFHxA, PFHpA, PFBS, PFPeS, PFHxS, and PFOS, showing a detection frequency very similar to that of the water grab samples analyzed in this study. EOF results ranged from 61 to 550 ng F sampler^–1^ and followed a similar pattern to the grab water samples from the groundwater and riverine deployments (Table S12). No fluorinated pharmaceutical compounds were detected in the passive samplers via a targeted analysis.

### Detection of Suspect Compounds

In groundwater, eight suspect compounds were identified in the unfiltered groundwater grab samples, while only seven of the same suspects were found in the passive sampler (Table S10). At the riverine site, there were six suspect PFAS found in passive samplers, while only three to four of the same compounds were identified in the unfiltered and filtered water (Table S10). Lastly, at site 3′s estuarine location, there were three of the same suspects found in both filtered and unfiltered water, while only one was detected in passive samplers (Table S10).

These observed differences between the grab sample and passive detection frequency are a potential limitation of this study and could be due to a myriad of factors. Shorter chain suspect compounds, like N-SPAmP-FBSA, may not be detected in passive samplers, despite detection in water grab samples, due to the low retention and membrane partitioning associated with shorter chain PFAS.^8^ Additionally, sampling rates were observed to be suppressed in this study in the presence of salinity, which may subsequently limit the detection of some suspect compounds in passive samplers versus water grab samples, particularly those collected from the estuary (Figure 1). However, in this study, salinity was not measured directly.

**Figure 1:**
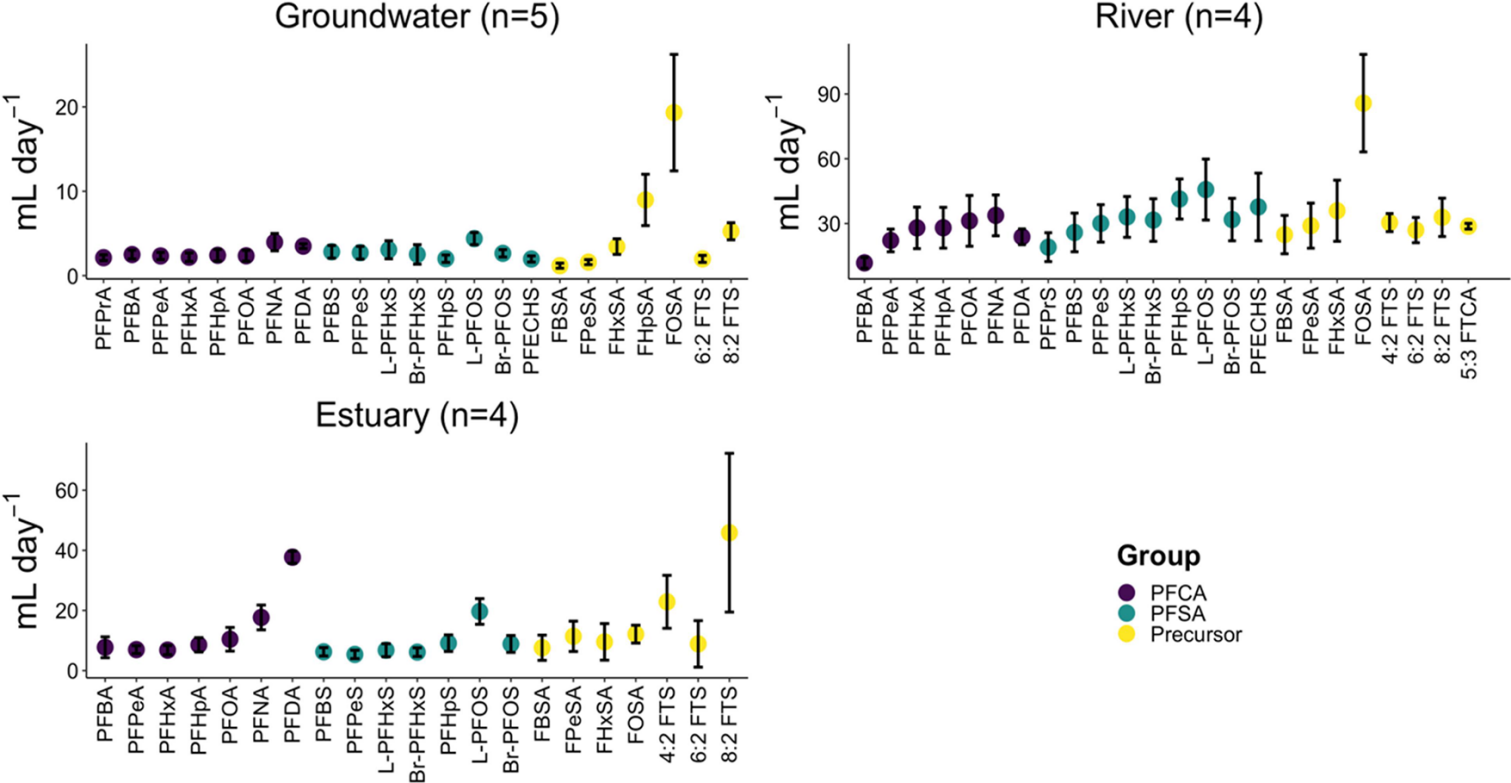
Field observed mean sampling rates for targeted PFAS in three water types. Error bars represent standard deviation among replicates.

### Target Compound Sampling Rates

Mean sampling rates (*R*_s,quant_) for 26 target compounds were calculated for each site where the compound was detected in both water and passive samplers (Figure 1 and Table S13). Mean *R*_s,quant_ ranged from 1.3 to 5.5 mL day^–1^ in the groundwater well near a fire training area (excluding the elevated FHpSA and FOSA) and were in good agreement with previously published values of 2.0–4.7 mL day^–1^ in groundwater. The FASA compounds FHpSA and FOSA were observed to have elevated sampling rates of 9.8–21 mL day^–1^.^9^ In the river, the average observed *R*_s_ for FOSA decreased from 84 to 48 mL day^–1^ using unfiltered water instead of filtered. This does not explain why FOSA *R*_s_ is elevated in the groundwater, however, as only unfiltered groundwater was used in this study. It is possible that FOSA bound to soil/sediments in the groundwater migrated into the passive sampler; however, a thorough evaluation of FOSA in sediment and soils would be required to further explore this hypothesis. The most likely explanation may be that FOSA and long chain FASA compounds exhibit high sampling rates, as they are not charged, leading to higher sorption to the hydrophobic, neutral exterior membrane of the HDPE MPT passive sampler.

The highest *R*_s,quant_ (12–86 mL day^–1^) were observed for the freshwater river (site 2). *R*_s,quant_ generally increased with the perfluorinated carbon chain length for PFCA, PFSA, FASA, and FTS compounds (Figure 1). At site 3, a tidal estuarine site, *R*_s,quant_ ranged from 5.4 to 46 mL day^–1^ in this study and also increased with perfluorinated carbon chain length. This trend of increasing *R*_s,quant_ with perfluorinated carbon chain length has been observed in a previous publication which reported 13–28 mL day^–1^ PFCA *R*_s_ across longer deployments (Figure 1).^10^ The mechanistic uptake of PFAS by the MPT passive sampler is influenced by partitioning of PFAS and their hydrophobic carbon–fluorine tail to the hydrophobic membrane of the MPT.^8^ Thus, PFAS uptake by the MPT is not governed solely by molecular diffusion. Past research has also highlighted the difference between groundwater and surface water sampling rates and suggested that sorption of PFAS on the MPT membrane may be impacted by the flow rate, as different partitioning values between membrane-water (*K*_mw_) were reported for flow and no flow conditions in laboratory tests.^8^

In this study, we calculated sampling rates for compounds without previous passive sampler calibration. The precursor compound, 5:3 FTCA, and the cyclical replacement compound, PFECHS, both exhibited targeted sampling rates similar to compounds of similar chain lengths (Figure 1). The cyclic 8-carbon PFECHS was similar to the 8-carbon compound PFOS, with average *R*_s,quant_ of 2.1 compared to PFOS 3.9 mL day^–1^ in the groundwater and 38 compared to PFOS 39 mL day^–1^ in the river. In the river site, 5:3 FTCA, an intermediate degradation product of 6:2 FTS, displayed uptake rates comparable to those of 6:2 FTS (29 vs 33 mL day^–1^) and PFHxA (29 vs 28 mL day^–1^), a terminal degradation product. The ultra short chain PFCA, PFPrA, was also observed for the first time to have minimal uptake compared to longer chain PFCAs in groundwater (Figure 1). For target sampling rate calibration of all 26 compounds reported in this study, see Table S13.

Lastly, there were small difference between sampling rates for linear versus branched isomers of PFHxS and PFOS with mean percent differences of 9 and 38%, as seen in Table S13. This suggests that the configuration of the carbon–fluorine chain may not be as influential on uptake as the length of the chain. This was further observed with the cyclical compound, perfluoroethylcyclohexane sulfonate (PFECHS), which displayed generally similar sampling rates to the 8-carbon chain of both PFOS isomers as well.

### Evaluation of EOF Sampling Rates

Mean EOF sampling rates (*R*_s,EOF_) were calculated as 7.1 ± 3.0 mL day^–1^ in groundwater and 47 ± 20 mL day^–1^ in the river (Figure 2). The observed *R*_s,EOF_ values for both sites were similar in magnitude to the average sampling rates of quantified target compounds at each respective site. Site 2 had larger uncertainty due to the presence of a likely outlier, as seen in Figure 2. It is possible that this outlier resulted from the EOF-deployed passive sampler being exposed to the air instead of water during falling water levels during the summertime deployment. The target and EOF passive sampler were deployed simultaneously but were free-floating in the water column at slightly staggered heights. Target MPT passive samplers codeployed with the EOF passive samplers showed consistency across replicates, suggesting that the targeted PFAS are not likely causing the low concentrations observed in this single outlier. The median *R*_s,EOF_ were calculated as 6.9 mL day^–1^ in the groundwater and 55 mL day^–1^ in the river.

**Figure 2:**
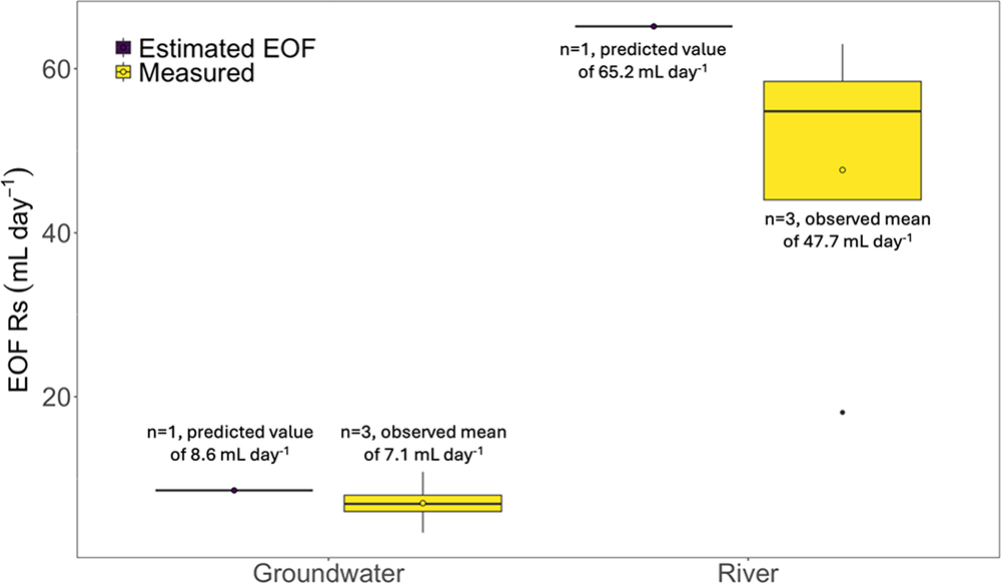
Predicted Versus Observed EOF Sampling Rates. Mean EOF sampling rates measured at each site (*n* = 3) are compared to the estimated EOF, referred to as *R*_S,Fnorm_ in the text (*n* = 1). Open circles represent the mean of the measured values, while the median is represented by the solid line across the box.

Good agreement is also observed when comparing the measured *R*_s,EOF_ to the estimated *R*_s,Fnorm_ (Figure 2), calculated from observed sampling rates of passive samplers codeployed next to the passive samplers that were measured for EOF; hence, it is also based on observational data. Site 1 had an estimate *R*_s,Fnorm_ of 8.4 mL day^–1^ (*n* = 1), and site 2 had an estimated *R*_s,Fnorm_ of 66 mL day^–1^. The length of the fully fluorinated chain emerged as a key parameter for estimating EOF sampling rates, as it affects both the fluorine content and the uptake rates into the sampler.

At site 1, previous studies have characterized the fluorine mass balance, indicating that the majority of the EOF reflects AFFF-derived PFAS, adding further justification to the estimation of the EOF sampling rate by using target and suspect compounds in eq 2.^21,48^ In contrast, site 2’s previous characterization revealed that only up to 60% of the EOF could be explained.^21,49^ The presence of fluorinated pharmaceuticals in the passive samplers was assessed to investigate whether they contributed to the previously observed unexplained fluorine, but no compounds were detected. The agreement between the estimated EOF sampling rates and observed EOF sampling rates suggests that either other fluorine-containing compounds are not readily retained by the passive sampler, which is the likely case for ultrashort chain PFAS due to low partitioning to the MPT membrane or are taken up at the same rate as these PFAS target and suspect compounds based on kinetics.^8^ If the unexplained fluorine was being taken up at greater rates than the PFAS and had a different sampling rate (for example, if *R*_s_ of fluorinated pharmaceuticals = 200 mL day^–1^) than the measured EOF sampling rate (58 mL day^–1^), it would likely further deviate from the estimated (66 mL day^–1^). These results indicate that conventional PFAS are the drivers of the EOF sampling rate due to their fluorine-rich structure (as fluorinated pharmaceuticals were not detected). This implies that passive sampling may be a valuable tool for EOF analysis providing a clearer picture of the scope of PFAS contamination at a given site. This is specifically true when determining long-term exposure of biota or communities to contaminated water, where bioaccumulation is better derived using time-averaged concentrations in place of discrete grab samples.^39,50^

### Different Approaches to Suspect Compound Sampling Rates

All *R*_s_ calculations resulted in the same trends of increasing *R*_s_ with chain length among PFCA, PFSA, FASA, and FTS groups (Table S14). The presence of salinity in the estuarine location inhibited passive sampler uptake of suspect compounds, as it did for target compounds. Due to similarities in the sampling rate of PFHxS and PFOS isomers, they are reported as the sum of both isomers in this section of the study.

In the groundwater, the various methods for calculating the sampling rate resulted in similar outcomes, with an average 9% difference between each approach and a maximum of 48% difference for the 24 PFAS evaluated (Figure 3). This suggests that in general, the method chosen to calculate *R*_s_ may not be overly influential on the outcome of groundwater deployments. We hypothesize that this insensitivity is likely due to lower particulate matter and matrix impacts in the groundwater system compared with surface water. Past research has identified that an environmental matrix, such as dissolved organic matter, can complicate suspect screening efforts.^42^ Groundwater sampling rates displayed in Figure 3 for FASA compounds display a muted increase with chain length compared to the target method results in Figure 1. However, the data presented in Figure 1 contain high levels of uncertainty, as depicted by the standard deviation error bars, likely from the presence of an outlier among the replicates.

**Figure 3:**
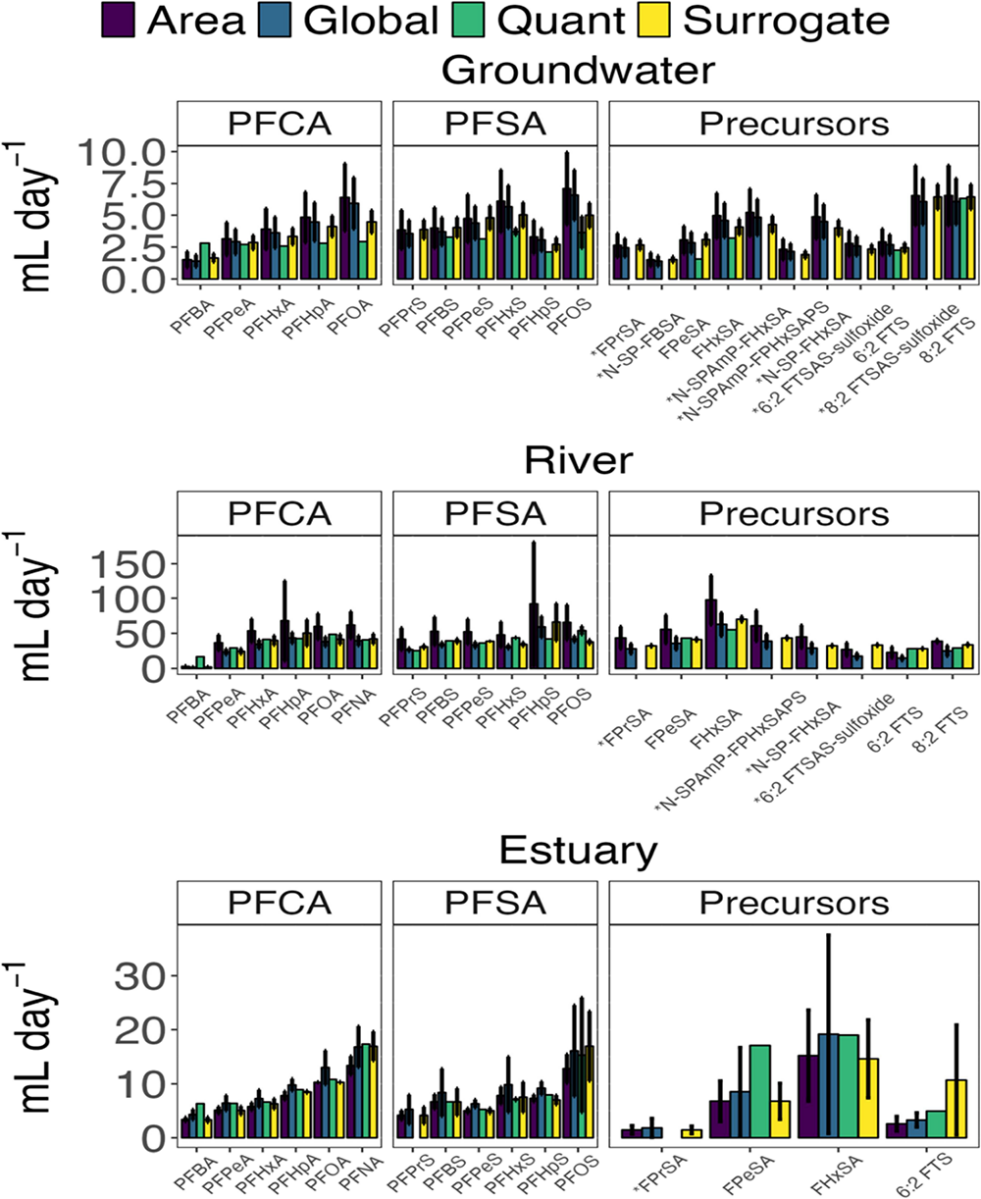
Comparison of discrete sampling rates of target and suspect compounds. Discrete sampling rates are reported for target and suspect compounds using both peak area and quantified data. For suspect compounds, quantified data are corrected as described in the text despite the lack of analytical standards or mass labeled surrogates. Sampling rates were calculated using unfiltered water, and error bars represent the uncertainty of the sampling rate for each class of PFAS (PFCA, PFSA, and FASA/FTS). FOSA results are not shown for scaling.

When suspect sampling rates were compared to known target compounds, the zwitterionic FTS precursors in groundwater, which, despite being detected using a different ionization mode than the 6:2 and 8:2 FTS target compounds, showed very similar sampling rates regardless of the method used to estimate *R*_s_ (Figure 3). Good agreement between the 6:2 zwitterionic FTS precursor and 6:2 FTS was also observed in the riverine samples, regardless of the calculation method (24 vs 26 mL day^–1^ on average, respectively).

However, a discrepancy was observed for the zwitterionic precursor N-SPAmP-FPHxSAPS (suspect), for which the estimated sampling rate differed by more than 10% from the nearest target compound’s surrogate-corrected sampling rate (FHxSA = 4.6 vs 2.2 mL day^–1^) at the groundwater location. This discrepancy may result from the different ionization modes, although this was not observed for the other zwitterionic FHxSA precursor N-SPAmP-FHxSA (Figure 3). The discrepancy for FHxSA precursor compounds was also observed in the river location, with a 46% lower average sampling rate for suspect N-SPAmP-FHxSA (zwitterionic) when compared to targeted FHxSA. There were larger discrepancies among raw, uncorrected peak areas (*R*_s,area_) and the other calculations evaluated for river water samplers (site 2), though overall good agreement (generally 10% difference from mean among calculation methods) was observed (Figure 3).

Overall, the *R*_s,surrogate_ approach for calculating sampling rates for unquantified suspect compounds seems to be the most applicable. *R*_s,surrogate_ routinely resulted in the lowest uncertainty of all sampling rate calculation methods considered here (Table S6). *R*_s,area_ regularly resulted in higher calculated sampling rates for both target and suspect compounds, as well as higher uncertainties, demonstrating this overly simplified approach is not applicable for estimating sampling rates for suspect and emerging PFAS. As discussed above, it is likely that the more complex surface water matrix complicates the use of the uncorrected *R*_s,area_ method. *R*_s,area_ is also likely to introduce bias from the lack of any mass labeled surrogate, which is typically used in targeted analysis as part of conversions from peak abundance to mass. Thus, the larger uncertainties, presented as error bars in Figure 3, are the likely result between the uncorrected areas and the surrogate-corrected areas. The use of *R*_s,area_ is only recommended for use within EOF comparison studies per the recommendation of current literature.^39^ *R*_s,global_ is also a poor choice for future work, as it generalizes across specific functional groups and PFAS families that otherwise have different chromatographic responses, as uncertainties for FASA were elevated when using *R*_s,global_ relative to other approaches. Due to the differences in PFAS functional groups and chemistry, it is preferable to use a representative surrogate of similar chemistry to avoid biasing the resulting sampling rate due to use of nonspecific, average chromatographic conditions. Following this proof-of-concept study, a more thorough understanding of how water properties (such as ions, pH, DOM, and particulates) may govern uptake and dictate the applicability of these equations should be investigated. This is especially important considering the use of the HLB sorbent in the MPT passive sampler design, which can bind a wide range of compounds aside from PFAS, including dissolved organic matter and other naturally occurring acids.

### Impact of Chain Length, Molecular Weight, and Retention Time

The importance of chain length, with partitioning to the HDPE membrane of the MPT sampler driving uptake, has been explored in detail in laboratory and numerical modeling studies, which support the conclusions of this study.^8^ In contrast, the positive relationship between the sampling rate and molecular weight only holds up to ∼500 g mol^–1^ (Figure S2). For example, large suspect compounds such as 6:2 FTSAS-sulfoxide and SPAmP-FHxSA, present at sites 1 and 2, have molecular weights that are 41 and 52% larger than their 6:2 FTS and FHxSA homologues. However, they have identical or slightly lower *R*_s,area_ and *R*_s,quant_ compared to their homologues (see Figure 3). This suggests that the fluorinated chain length and functional group may be more important in predicting sampling rate and uptake performance than overall molecular weight.

Figures 4 and S2 display linear regressions for modeling the sampling rate (*R*_s,surrogate_) based on fluorinated chain length and molecular weight, respectively. The suspect compounds display a better overall fit across all three sites when using fluorinated chain length as a proxy instead of molecular weight, as illustrated by the high molecular weight suspect compounds not following the established trend lines (Figures 4 and S2). These regressions were calculated using the *R*_s,surrogate_ of targeted compounds, while the suspects were overlaid on top to assess if they fit the same trends. When all PFAS types are grouped together, the groundwater sampling rates show less of an increase in sampling rate with chain length than was observed within each group in Figure 3, with p values >0.05 suggesting there is no significant linear relationship (Figures 3 and 4). Groundwater results did not result in p values >0.05 in any of the linear regressions performed on target compound relationships (Figures 4, S2 and S3), demonstrating that sampling rates did not depend on chain length in this low flow environment compared to the high flow river and estuary deployments (*p* < 0.05 for all).

**Figure 4:**
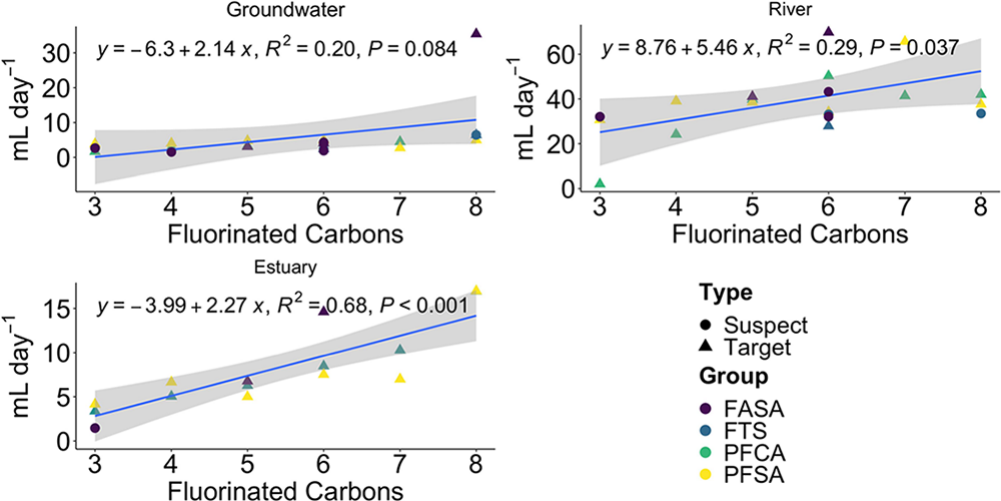
Trends in sampling rates (*R*_s,surrogate_) across fluorinated chain length of target and suspect compounds for the three study sites (1) groundwater, (2) river, and (3) estuarine. Regression is performed only on target compounds with suspect compounds overlaid on top. Standard error of the regression is shaded in gray.

Current approaches have focused on the use of a global correction, where suspect peak areas are normalized to the average peak area of all internal standards added.^41,46^ While that approach agrees well with other methods considered in this study, it is worth noting that many of the suspect compounds in this study are structurally similar to the PFSA, FTS, and FASA surrogates in the target method. Future work will need to test the applicability of fluorinated chain length-based approaches for estimating *R*_s_ with more diverse PFAS chemical structures.^51^

Comparison of chromatographic retention times with *R*_s,surrogate_ showed a similar if not better correlation than with chain length, with suspect precursors showing retention times similar to those of their terminal end products (Figure S3). Retention time alone cannot accurately predict the sampling rate across all PFAS, but the correlations imply that the behavior/sampling rate of suspect compounds is strongly linked to the sampling rate of their terminal products of similar chain length and functional group (Figure S3).

These findings suggest that overall, factors like fluorinated chain length and similarity in chemistry to terminal PFAS may be what primarily drives suspect compound uptake by the passive sampler. Thus, the best approach for deducing sampling rates for PFAS without analytical standards may be using those for PFAS of a similar chemistry. Increasing binding of PFAS with increasing chain length was also observed for serum proteins, before plateauing as was observed in this study of sampling rates.^52^ These observations imply that the uptake of certain precursor compounds into the MPT passive samplers may mimic biological uptake.

### Identifying Three Distinct Sampling Regimes

On average, there was a 2.5-fold decrease in sampling rates between sites 2 (fresh) and 3 (saline). While salinity was not directly measured in this study, typically salinity for a neighboring river (Childs River) entrance into Waquoit Bay is 25.9 PSU.^53^ This observation can help explain the discrepancies reported previously for deployments in fresh versus estuarine and coastal waters.^8,10^ Salinity can increase or decrease the sampling rate, based on the specific organic contaminant’s chemistry and interactions in the water column.^54^ In general, organic contaminants can form complexes with polyvalent cations, which can lead to reduced uptake by passive samplers, and the anionic nature of many of the PFAS in this study suggests they may be susceptible to forming complexes.^54^

However, the impact of salinity on PFAS partitioning in the environment has also been previously characterized and increases in salinity led to higher concentrations of PFAS at the air–water interface, which would impact grab samples more than passive samplers, as the latter are exposed in the water for weeks.^55^ However, past laboratory and modeling studies have evaluated the impacts of biofouling, temperature, and flow rate on the MPT passive sampler that support the results of this study.^8^ Investigation of the impact of environmental parameters on the MPT should be studied further.

### Implications

This study showed the importance of perfluorinated carbon chain length in determining sampling rates for EOF, target, and suspect compounds by a MPT passive sampler. Suspect compound sampling rates were generally similar to those of targeted compounds with similar fluorinated chain length and functional group (mean percent difference = 16%). Likewise, field observed EOF sampling rates were well predicted by normalizing individual PFAS sampling rates to the number of fluorines and summing all compounds present (<20% difference from the median). We suggest that these sampling rates can be applied to derive EOF and suspect compound sampling rates in groundwater and surface waters, with additional method development needed for EOF in passive samplers deployed in saline waters.

The proposed sampling rates for the suspect compounds provide important insights into their chemical fate and transport in aqueous media. The sampling rate is governed by a complex mix of compound diffusivity, MPT membrane-water partitioning, and sorbent-water partitioning.^8^ Our results imply that some suspect compounds display diffusive and partitioning behavior similar to that of target compounds for the MPT passive sampler. This is increasingly relevant, as uptake of FASA precursors and other AFFF-derived PFAS has been observed in biota, with some exhibiting toxicity and high bioaccumulation factors, exacerbating the need for enhanced PFAS detection tools for stakeholders.^34,50,56–58^

With the list of PFAS of concern growing but the availability of matching analytical standards lagging, this study suggests that simple estimates of suspect compounds’ sampling rates (*R*_s,surrogate_) can be derived. The same passive sampler design has already shown the ability to help identify new PFAS through nontarget analysis (nonquantitative).^59^ Lastly, our understanding of the MPT passive sampler uptake kinetics has improved, showing that salt and fresh surface water deployments will require the use of different sampling rates, regardless of the MPT’s previously validated resistance to flow and biofouling.^8^ This will guide future attempts to improve calibration through numerical modeling and parametrization of MPT passive sampler uptake. Our study’s proposed EOF and suspect compound sampling rates greatly expand the ability of the MPT passive sampler to determine time-weighted average measurements for monitoring and detection in fate and transport, forensic, and bioaccumulation studies. This study outlines a clear path forward for utilizing the MPT passive sampler for a growing list of PFAS of concern.

## Supplementary Material





## Notes

Additional details on methods and results including instrumental parameters, quality control, method development, and data handling, and additional results (PDF)
Data tables relevant to quality control, method development, instrumental parameters, and results (XLSX)
