# Supplementary material for: Perfluorinated Carbon Chain Length Drives Uptake of Diverse Per- and Polyfluoroalkyl Substances in Field-Deployed Passive Samplers
Source: ACS ES T Water. 2025 Mar 7;5(4):1803–13. doi: 10.1021/acsestwater.4c01164 (PMC12393212; doi:10.1021/acsestwater.4c01164)
Supplement: Supplementary file 1 [file ew4c01164_si_001.pdf]

Supplemental Information

Perfluorinated carbon chain length drives uptake of diverse Per- and polyfluoroalkyl substances in field-deployed passive samplers

By

Matthew Dunn[1], Jitka Becanova[1], Simon Vojta[1], Heidi Pickard[2], Rainer Lohmann[1]

1. Graduate School of Oceanography, University of Rhode Island, Narragansett, RI 02882, USA
2. Harvard John A. Paulson School of Engineering and Applied Sciences, Harvard University, Cambridge MA, 02138, USA

Corresponding author: Rainer Lohmann, rlohmann@uri.edu

Additional Instrumental Analysis Details. Concentrated methanol extract was reconstituted with 10mM ammonium acetate in water to reach final ratio of 40/60 (methanol/water). A Phenomenex Gemini 3um C18 110Å 50x2mm LC analytical column preceded with a Phenomenex SecurityGuard cartridge was used for the analyte separation. Another Phenomenex Gemini 5um C18 110 Å 50x4.6mm LC analytical column was used to delay the PFASs instrumental contribution. The aqueous mobile phase (MPA) was 10mM ammonium acetate in water and the organic mobile phase (MPB) was 10mM ammonium acetate in methanol. LC parameters were set as follows: flow 0.3mL/min, injection 20uL, column oven 45°C. Solvent gradient of MPB gradually increased from 40% to 80% (1 to 5.5 min), 80% to 100% (5.5 to 7 min), then hold for one minute and finally drop to 40% (8 to 8.5 min) and hold for another 6.5 minutes.

Additional SPE Filtration Details. Splitting and filtration of grab samples prior to SPE analysis was performed investigate if removing the particulate phase from the water would result in different overall PFAS concentrations for both target and non-target analysis. Additionally, the use of a glass fiber filter could sorb neutral PFAS precursors that are not as water soluble, which would reflect in different concentrations for these compounds between the samples. And lastly, by filtering the water and extracting the dissolved phase, we thus remove additional matrix differences between the water and passive sampler that may impact uncertainty of the semi-quantitative suspect screening.[1] Both the SPE cartridge and tube passive sampler become a column of sorbent with only the dissolved phase of PFAS bound. Filtration was not performed on grab samples from site 1 because groundwater generally has low particulate matter. Unfiltered 500 mL samples were extracted for EOF in accordance with prior work.[2,3] Matrix spike recoveries for this method are reported in Table S3.

Additional Sampling Rate Details. Typically, sampling rates have been numerically modeled, but this requires detailed information on diffusivity and partitioning to membrane and sorbent.[4–7] For the specific tube passive sampler design in this study, only 9 PFAS have measured sampling rates in the lab and field or predicted by models.[4] Thus, these previously used modeling efforts were not applied to compute sampling rates for suspect compounds without established physio-chemical parameters such as their aqueous diffusive coefficient or sorbent-water partitioning.

Quality Assurance and Quality Control Details for Target and Suspect Analysis. Blank correction for target analysis of passive and active samples was done by subtracting the average blank concentration from the sample concentration. If the average blank exceeded 30% of the native concentration of a compound in a given sample, that compound was not reported. If blank samples had non-detects no correct was performed. However, for method detection limit (MDL) calculations, non-detects were replaced by one half of the instrumental detection limit (IDL) was used. IDLs were calculated as the average concentration at which the calibration curves' samples signal to noise ratio (S/N) was 10. Method detection limits were then calculated as the median of blank concentrations plus three times the standard deviation of these blanks.

Recoveries were calculated as the response of the surrogate measured in the concentrated sample divided by the response of the surrogate in the calibration curve, with both spiked to a goal concentration of 4 ng mL^{-1}. For active grab sample quality control, targeted analysis solid phase extraction efficiency was evaluated using matrix spikes. 50 mL of tap water was spiked with a known quantity of analytical PFAS standards, including 11 of the 12 compounds

discussed in this study (Table S3). A matrix blank showed no detection of PFAS in the tap water volume used for these experiments. The results of this matrix spike can be found in Table S4 and show good recovery (84-110%). Suspect screening was also performed on a single passive sampler field blank from the river deployments (site 2). Only PFOS, PFOA, and PFHxS were identified in the passive sampler at low levels, in accordance with the low levels reported for the targeted analysis of this field blank sample (no detect for PFHxS and PFOS, ~ 1 ng $sampler^{-1}$ for PFOA). Thus, no correction of the suspect compound areas were made for the passive sampler data.

Quality Assurance and Quality Control Details For EOF Analysis. For EOF analysis at Harvard University, a ceramic boat without sample (boat blank) was analyzed twice between each set of duplicate sample injections to determine background fluorine (F) levels between sample injections. Samples were blank corrected using the peak areas of the boat blanks run before and after each set of injections. Extraction blanks using DI water for grab sample SPE extraction (n=2) and blank passive samplers for passive sampler SPE extraction (n=2) were used to blank correct sample concentrations and to determine the LOD, which was calculated as the average plus three times the standard deviation of duplicate injections of extraction blanks. The extraction LOD for grab samples was 126 ng F/mL and for passive samplers was 98 ng F/mL. Extraction blanks were all <LOD but would have accounted for 14-40% of EOF in the grab samples and 4-27% of EOF in passive samplers if average extraction blank concentrations were not subtracted from the samples. Individual sample MDLs were calculated based on the extraction LOD multiplied by each sample's dilution factor based on sample and extract volumes. Sample MDLs for grab samples ranged between 0.117 – 1.69 ng F/mL. Sample MDLs

for passive samplers ranged between 44.7 – 65.9 ng F/passive. Sample concentrations were determined from the average peak areas of duplicate injections using a 12-point calibration curve (R^2>0.998) of PFOA as F^- equivalents in LC-MS MeOH from 50.4 to 1000.76 μg F/L. Relative standard deviation (RSD) between duplicate injections for samples ranged between 0 - 42% but averaged 8% (median of 4%). Quality control points (n=4) were included after every 12 samples and had a variance of <15%.

For EOF grab samples, all methodology and quality control was based on methods described in previous literature.[3,8] For the novel application of EOF analysis on passive samplers, OF recovery was assessed separately using a 995 ng F/mL PFAS mixture (Wellington PFAC-24PAR) spiked into blank passive samplers (n=3). OF recovery was assessed on passive samplers extracted with and without the IF rinse step to assess if the IF rinse impacted OF recovery in the passive samplers. The IF rinse consisted of 6-9 mL of 0.01 v/v ammonium hydroxide in Milli-Q water. OF recovery in passive samplers (n=3) without the IF rinse step yielded a 82-91% recovery. OF recovery in passive samplers (n=3) with the IF rinse step yielded a 75-92% recovery. Similar recovery ranges indicates the IF rinse step does not have impact on OF recovery. IF removal efficiency was assessed separately using a 1000 ng F/mL sodium fluoride spiked into blank passive samplers (n=3). IF removal was also assessed on passive samplers extracted with and without the IF rinse step to assess how much of an impact the IF rinse has on removal inorganic fluorine spiked into passive samplers. IF removal in passive samplers (n=3) without the IF rinse step yielded a 89-100% removal. IF removal in passive samplers (n=3) with the IF rinse step yielded a 98-99% removal. All IF spiked samples were <MDL indicating efficient removal with the IF rinse yielding less variability in the removal efficiency between n=3 samples assessed.

EOF concentrations > MDL for grab samples and passive samplers deployed in groundwater (n=6) and river water (n=8) are reported in this study. EOF measurements for estuarine samples were excluded from interpretation in this study due to high chloride content impacting fluoride quantification. Further method development for EOF analysis of samples with high chloride content are needed.

Additional Details on Calculation of Suspect Uncertainty. The approach for estimating sampling rate of suspect compounds is a ratio-based calculation, not a semi-quantitative approach where accurate masses of suspect compounds need to be carefully identified. Instead, the mass, or in this case area abundance, ratio between the passive sampler and water grab results are used. Thus, there is less concern about the exact magnitude of the suspect compound abundance, assuming that any uncertainty in the LC-QTOF measurement is at least consistent across each compound. I.e., whatever uncertainty is associated with N-SP-FHxSA is biased in the same manner every time it is measured. To display this approach, the use of target compounds with a known LC-QTOF performance can be used to validate this approach if we treat them like suspects (i.e. using unadjusted peak area abundance).

$$R_{s,Area} = \frac{Area_{PFHxA,\,Passive}}{Area_{PFHxA,\,Water} * deployment\ time} \quad (eqn\ 1a)$$

Equation 1 display this approach, where the peak area of PFHxA can be used to estimate the sampling rate. This same approach was applied to suspect compounds, as it is assumed that any uncertainty in the passive (numerator) or water grab sample (denominator) area abundance measured for suspect compounds was the same.

$$R_{s,Area} = \frac{Area_{N-SP-FHxSA,\,Passive}}{Area_{N-SP-FHxSA,\,Water} * deployment\ time}\ (eqn\ 1b)$$

This same equation and approach were applied when using areas that were corrected using a mass labeled surrogate area of similar chemistry, or a global average of all surrogate areas.

$$PFHxA\ Area_{Surrogate} = \frac{Area_{PFHxA}}{Area_{m-PFHxA}}\ (eqn\ 2a)$$

Equation 2a displays this approach, with m-PFHxA indicating mass labeled surrogate. As an example of how suspect compounds may have been assigned a surrogate, see the example in equation 2b. In this case, the compound with the most similar chain length that was present as a mass labelled surrogate was PFHxS, though investigations were also done to use a common with the most similar functional group, FOSA. As previously mentioned, using a global average of mass labeled surrogate areas in the denominator was also investigated.

$$N - SP - FHxSA\ Area_{Surrogate} = \frac{Area_{N-SP-FHxSA}}{Area_{m-PFHxS}}\ (eqn\ 2b)$$

With quantified sampling rates already available for compounds like PFHxA or PFHxS from the targeted analysis, an approximation of how uncertain the $R_{s,area}$, $R_{s,surrogate}$, or $R_{s,global}$ values could now be calculated. This was performed by calculating the percent difference between the $R_{s,area}$, $R_{s,surrogate}$, or $R_{s,global}$ values from the quantified sampling rates ($R_{s,quant}$) for all target compounds.

$$\%\,Uncertainty = Abs\left[\frac{PFHxA\ R_{s,quant} - PFHxA\ R_{s,area}}{PFHxA\ R_{s,quant}}\right] * 100$$

This was performed across all compounds and the average percent uncertainty, or difference from the quantified known value, was then taken for a series of groups of representative compounds (PFCA, PFSA, FTS/FASA). For example, the average percent uncertainty at site 1 for all PFCA compounds ranged from 20-42% depending on which approach you used ($R_{s,area}$ vs $R_{s,surrogate}$ vs $R_{s,global}$). Suspect compounds were then assigned the uncertainty of the group they shared the most alike chemistry with, which in this case was PFSA or FTS/FASA compounds from the targeted method. See table S6 for a thorough breakdown of all values.

For example, if PFOS shows an $R_{s,quant}$ of 8.5 mL day^{-1} from field deployments, yet the $R_{s,area}$ approach reports a value of 13 mL day^{-1}, we can assume the potential bias, or uncertainty, associated with calculating $R_{s,area}$ value is nearly 50%. The suspect compounds included in this study are generally either PFSA, FTS, or FASA precursors that have a similar chain length and chemistry to PFSA, FTS, or FASA compounds in the targeted method applied here. Thus, it is assumed that their bias or uncertainty will be similar to other compounds with similar functional group, if all compounds, targeted or suspect, are calculated using the same methods outlined in Table 1. For example, this means that the uncertainty applied to N-SP-FHxSA is derived from the uncertainty observed from FHxSA. This is a broad assumption because of the difference in ionization modes between some of these precursors and the FASA and FTS compounds discussed in this study, however we present this work as a proof of concept.

Comparison of Filtered versus Unfiltered PFAS water concentrations. To further expand on the impact of filtration, figure S1 shows a comparison of results for filtered and

unfiltered water from sites 2 and 3. Both sites 2 (river water) and 3 (estuarine water) displayed slopes approaching 1.0 when comparing filtered and unfiltered water that was fully quantified using targeted analysis (1.02 and 0.942 respectively). This shows good agreement between both water conditions, suggesting that the impact of the environmental matrix on uncertainty is minimized using a targeted method. However, the river site, site 2, shows a less strong fit between unfiltered and filtered peak areas, which are not corrected with internal standards or calibration curves (slope = 0.701) (Fig S1). In the context of calibrating the passive sampler for compounds identified through suspect screening, it is crucial to employ water grab filtration. This practice enhances the consistency between the data derived from solid-phase extraction applied to filtered water samples and data obtained via passive samplers, as both methods exclusively extract dissolved PFAS. In terms of filtration impacts on suspect screening, there was generally similar results between sites 2 and 3. Filtered water at site 2 had 6 suspect compounds identified versus only 5 in the unfiltered water. Site 3 had the same 5 suspect compounds detected in both filtered and unfiltered water.

Determination of water volume sampled by passives. Using the average sampling rate of all compounds found at each site, we calculated the volume of water each tube passively sampled over the 7 or 14-day deployments. Passive samplers extracted an average of 26 ($\pm$ 26) mL of groundwater at site 1, about 450 ($\pm$ 190) mL of river water at site 2, and 180 ($\pm$ 150) mL of estuarine water at site 3. These volumes are similar to the volumes of grab water samples extracted using SPE for these sites (50-500 mL).

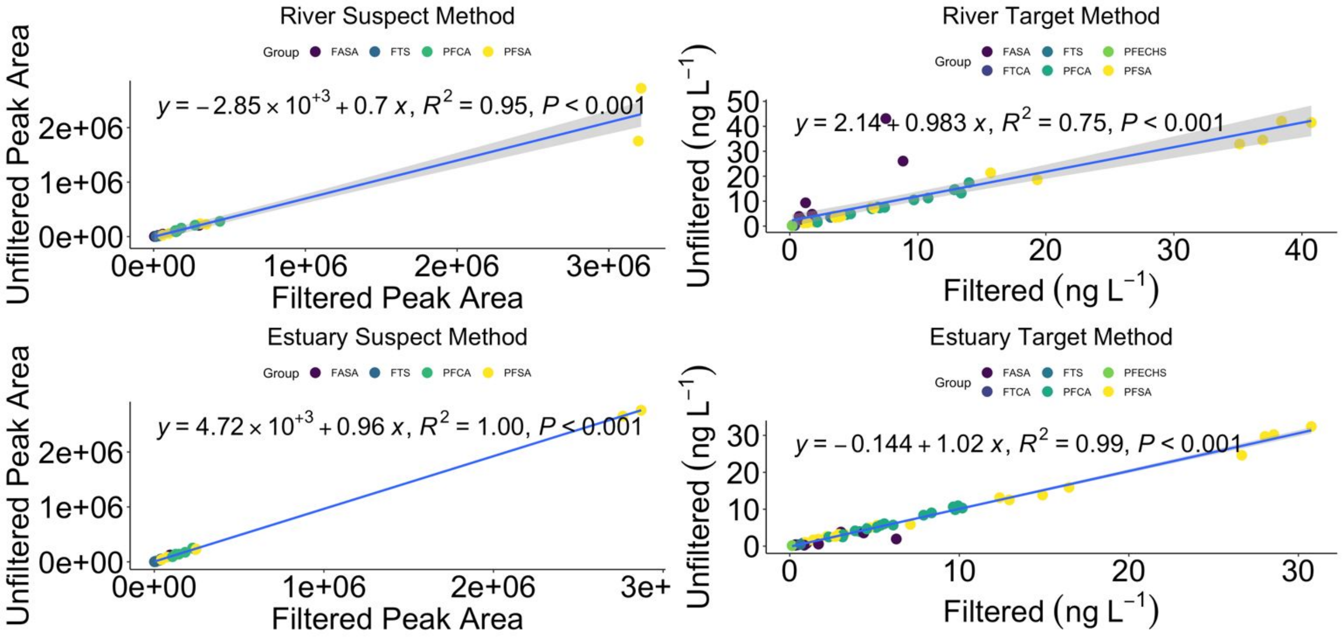

Figure S1. Comparison of PFAS concentrations in filtered vs unfiltered water derived by both peak area and quantified concentration. Standard error of the regression is shaded in grey.

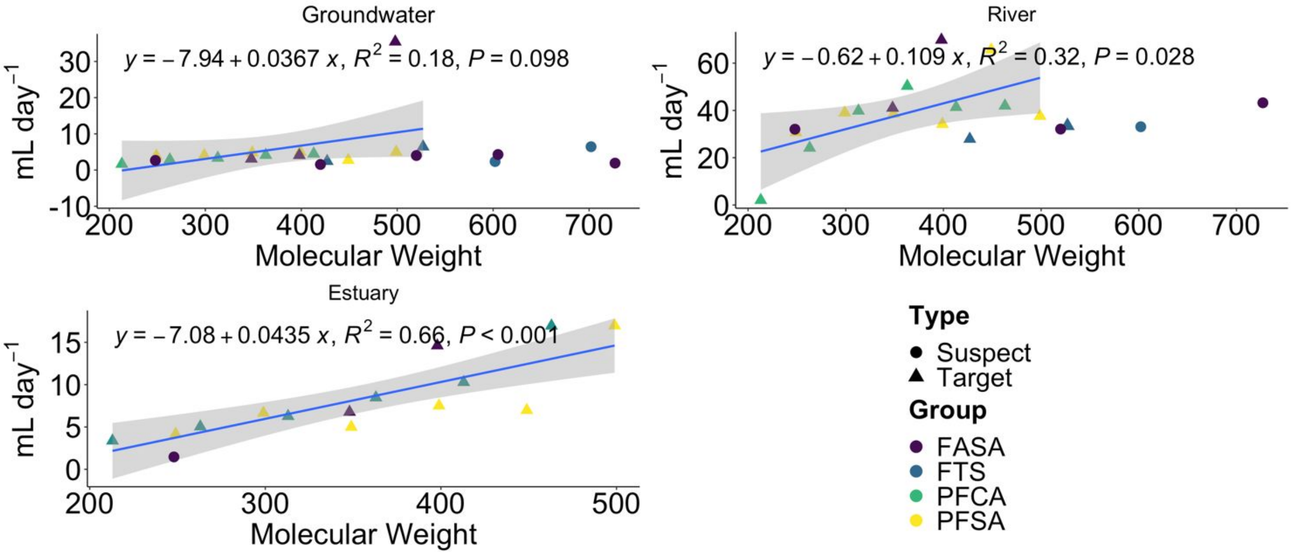

Figure S2. PFAS sampling rates ($R_{s,surrogate}$) as a function of molecular weight of target and suspect compounds for sites 1, 2 and 3. Regression is performed only on target compounds, with suspect compounds overlayed on top. Standard error of the regression is shaded in grey.

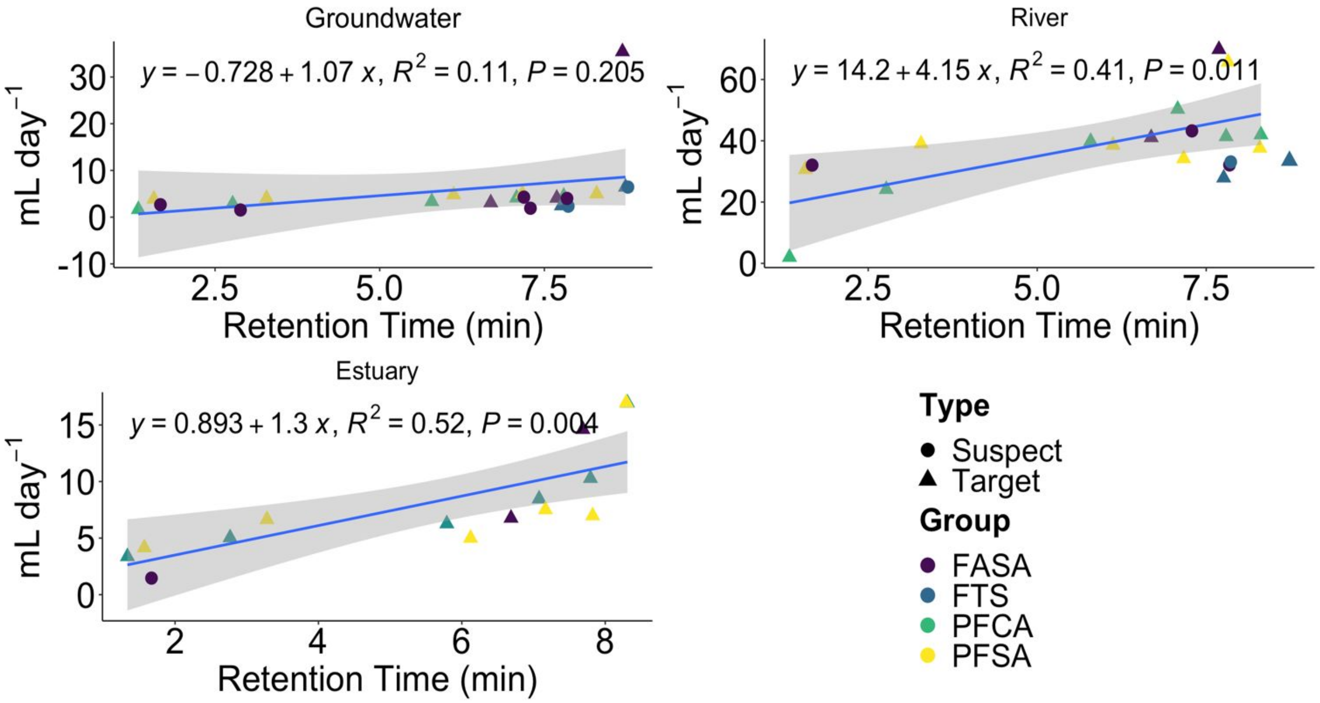

Figure S3. PFAS sampling rates ($R_{s,surrogate}$) as a function of chromatographic retention time of target and suspect compounds for the 3 sites. Regression is performed only on target compounds, with suspect compounds overlayed on top. Standard error of the regression is shaded in grey.

References

(1) Dickman, R. A.; Pu, S.; Sayre-Smith, N.; McCord, J.; Sobus, J.; Aga, D. S. Towards Quantification Without Standards: Impacts of Environmental Matrices on the Solubilities and Ionization Efficiencies of Per- and Polyfluoroalkyl Substances (PFAS).

(2) Ruyle, B. J.; Thackray, C. P.; McCord, J. P.; Strynar, M. J.; Mauge-Lewis, K. A.; Fenton, S. E.; Sunderland, E. M. Reconstructing the Composition of Per- And Polyfluoroalkyl Substances in Contemporary Aqueous Film-Forming Foams. *Environ. Sci. Technol. Lett.* **2020**. https://doi.org/10.1021/acs.estlett.0c00798.

(3) Ruyle, B. J.; Pickard, H. M.; LeBlanc, D. R.; Tokranov, A. K.; Thackray, C. P.; Hu, X. C.; Vecitis, C. D.; Sunderland, E. M. Isolating the AFFF Signature in Coastal Watersheds Using Oxidizable PFAS Precursors and Unexplained Organofluorine. *Environ. Sci. Technol.* **2021**, *55* (6), 3686–3695. https://doi.org/10.1021/acs.est.0c07296.

(4) Dunn, M.; Becanova, J.; Snook, J.; Ruyle, B.; Lohmann, R. Calibration of Perfluorinated Alkyl Acid Uptake Rates by a Tube Passive Sampler in Water. *ACS EST Water* **2023**, acsestwater.2c00384. https://doi.org/10.1021/acsestwater.2c00384.

(5) Booij, K. Passive Sampler Exchange Kinetics in Large and Small Water Volumes under Mixed Rate Control by Sorbent and Water Boundary Layer. *Environ. Toxicol. Chem.* **2021**, etc.4989. https://doi.org/10.1002/etc.4989.

(6) Booij, K.; Chen, S.; Trask, J. R. POCIS Calibration for Organic Compound Sampling in Small Headwater Streams. *Environ. Toxicol. Chem.* **2020**, *39* (7), 1334–1342. https://doi.org/10.1002/etc.4731.

(7) Endo, S.; Matsuura, Y. Mechanistic Model Describing the Uptake of Chemicals by Aquatic Integrative Samplers: Comparison to Data and Implications for Improved Sampler Con Fi Gurations¨. *Environ. Sci. Technol.* **2019**, *53*. https://doi.org/10.1021/acs.est.8b06225.

(8) Ruyle, B. J.; Pickard, H. M.; Schultes, L.; Fredriksson, F.; Heffernan, A. L.; Knappe, D. R. U.; Lord, H. L.; Meng, P.; Mills, M. A.; Ndungu, K.; Roesch, P.; Rundberget, J. T.; Tettenhorst, D. R.; Van Buren, J.; Vogel, C.; Westerman, D. C.; Yeung, L. W. Y.; Sunderland, E. M. Interlaboratory Comparison of Extractable Organofluorine Measurements in Groundwater and Eel (*Anguilla Rostrata*): Recommendations for Methods Standardization. *Environ. Sci. Technol.* **2023**, acs.est.3c04560. https://doi.org/10.1021/acs.est.3c04560.
